# Perceived School Performance, Life Satisfaction, and Hopelessness: A 4-Year Longitudinal Study of Adolescents in Hong Kong

**DOI:** 10.1007/s11205-015-0904-y

**Published:** 2015-02-15

**Authors:** Daniel T. L. Shek, Xiang Li

**Affiliations:** 1 Department of Applied Social Sciences, The Hong Kong Polytechnic University, Hunghom, Hong Kong; 2 Psychological Studies Academic Group, National Institute of Education, Nanyang Technological University, Singapore, Singapore

**Keywords:** Perceived school performance, Life satisfaction, Hopelessness, Chinese adolescents, Longitudinal design

## Abstract

This 4-year longitudinal study examined the perceived school performance, life satisfaction, and hopelessness of Chinese adolescents in Hong Kong. Over the period of the study, perceived school performance and life satisfaction decreased, whereas adolescent hopelessness increased. Consistent with our predictions, a positive relationship between perceived school performance and life satisfaction, a negative relationship between life satisfaction and hopelessness, and a negative relationship between perceived school performance and hopelessness were found. Structural equation modeling further showed that life satisfaction functioned as a mediator in the relationship between perceived school performance and hopelessness. The findings underscore the role of perceived school performance in adolescent well-being and suggest that promoting life satisfaction is a possible way of reducing adolescent hopelessness.

## Introduction

### The Importance of School Performance in Chinese Society

Chinese society’s strong emphasis on school success is well known around the world. There is a traditional Chinese belief that “to be a scholar is to be the top of society” (*xue er you ze shi, 學而優則仕*). Driven by this thinking, Chinese students are expected to study hard and achieve academic excellence (Shek and Chan 1999). Such beliefs have created a highly selective and competitive education system in Hong Kong (Kwan 2010), which drives students to study hard and perform well to achieve good school results in the harsh public examination system. Under the influence of Confucianism and related Chinese philosophies, academic performance and school conduct are two closely related aspects of school performance (Ali 2011). In addition to achieving good grades, Chinese children are expected to have good school conduct (Li and Fung 2014).

In Hong Kong, students experience fierce school competition from early childhood, as their performance at each learning stage determines whether they will be accepted to study at a distinguished school in the next stage. Among different age groups, secondary school students experience the greatest stress because they must pass the Hong Kong Diploma of Secondary Education Examination to get into university, which is a conventional route to future success. In particular, the 3-3-4 scheme introduced in the academic structure reform (i.e., the new senior secondary school curriculum) implemented in Hong Kong in 2006 has increased the burden on students. Under the new system, students have only three instead of 4 years of senior secondary education to prepare for the university entrance examination. Students must work much harder to adapt to this new initiative and achieve academic excellence in a shorter time. Against this background, students are very likely to suffer from more emotional problems because of their stressful school experiences (Au and Watkins 1997; Long et al. 2012).

### Perceived School Performance and Adolescent Hopelessness

Researchers have defined hope as an individual’s overall perception that he or she can meet his or her goals (e.g., Snyder et al. 1991). Other researchers have defined hopelessness as a negative expectation toward oneself and the future (e.g., McLaughlin et al. 1996). Individual hopelessness may be reflected in unfavorable expectations about one’s future life (Beck et al. 1974). Abramson et al. (1989, p. 359) defined hopelessness as “an expectation that highly desired outcomes will not occur or that highly aversive outcomes will occur coupled with an expectation that no response in one’s repertoire will change the likelihood of occurrence of these outcomes.” Researchers have examined whether hope changes during adolescence. McKnight et al. (2002) reported that positive affect decreased over time in adolescents. However, Bolland (2003) found that age was not related to a sense of hopelessness in male adolescents, whereas age and hopelessness were negatively correlated for female adolescents. In view of the inconsistent research findings, there is a need to further examine this issue.

Daily events or chronic stress have considerable influence on individuals’ lives and, in particular, stressful life events cause intrapersonal problems for adolescents (McKnight et al. 2002). School performance is one of the precursors of hopelessness because school learning is the major developmental task of adolescents in Chinese culture (Huang 2014). As school performance is almost the sole standard for predicting future success, school performance is of great importance for Chinese adolescents and those surrounding them, such as parents and teachers (Huang 2014).

Adolescents’ lives are filled with a variety of positive and negative stressors (McKnight et al. 2002), the most important of which may be school performance because experiences at school are the most typical and influential sources of stress for adolescents (Ash and Huebner 2001). Perceptions of personal academic capabilities and academic self-image influence student well-being (Van Petegem et al. 2007) and school conduct is positively related to perceived academic performance (Roeser and Eccles 1998). If students worry about and have negative perceptions of their school performance, it will create negative affect (Long et al. 2012). In Chinese societies, adolescents usually display hopelessness if their school performance is unsatisfactory (Shek and Li 2014).

Poor perceptions of academic performance and school conduct are related to depressive feelings and negative psychological adjustment (Roeser and Eccles 1998). Studies have documented the relationship between perceived school performance and hopelessness. Typically, successful school experiences help students to develop a positive sense of well-being, whereas school failures not only increase the feeling of hopelessness, but also contribute to poorer perceptions of their academic self-image and school performance (Au and Watkins 1997). Pekrun et al. (2009) also found that scholastic ability was negatively related to hopelessness. Perceived school performance has been related to self-esteem and locus of control, and found to predict students’ emotions and behavioral problems such as depression and suicide attempts (Richardson et al. 2005). Some studies have found that students who perceive that they have little control over their examination performance are prone to high levels of hopelessness and unhappiness (e.g., Burić and Sorić 2012). It can be argued that when students feel incompetent in school, they can easily lose confidence and feel hopeless about moving forward into the future. Chinese students may be particularly likely to feel hopeless if they have poor perceptions of their school performance because of the strong emphasis on academic achievement in Chinese culture.

### Life Satisfaction as a Mediator

As an important construct of positive psychology, life satisfaction has been regarded as a stable indicator of personal well-being (Gilman et al. 2000) and psychological development (Goldbeck et al. 2007) in adolescence. Life satisfaction is a global cognitive evaluation of an individual’s life as a whole (Suldo and Huebner 2004). McCullough et al. (2000) found that the majority of adolescents in secondary schools had moderately high levels of life satisfaction. Some scholars have argued that age does not influence adolescent life satisfaction and the trend of life satisfaction is moderately stable in adolescence (e.g., Ash and Huebner 2001; Suldo and Huebner 2004). However, Gilman and Huebner (2003) argued that life satisfaction as a developmental phenomenon is not static but volatile for adolescents, because adolescence is one of the most difficult periods in human development, with intense cognitive, emotional, and social changes (Arnett 1999). Goldbeck et al. (2007) found that adolescent life satisfaction decreased over time in many countries, including Germany, Australia, and Poland. Michel et al. (2009) found that adolescents generally had a poorer quality of life than children, based on data from 12 European countries, including the United Kingdom, France, the Netherlands, and Sweden.

Ash and Huebner (2001) proposed that life experience and life satisfaction have a transactional relationship. For example, positive daily events lead to high life satisfaction (McCullough et al. 2000) and negative perceptions of school experience usually lead to low life satisfaction (Gilman and Huebner 2006). Empirically, Chow (2008) found a positive relationship between academic achievement and life satisfaction in adolescents. Suldo et al. (2008) found that problematic school behavior was associated with low levels of school life satisfaction and global life satisfaction.

In addition to the positive relationship between perceived school performance and life satisfaction, life satisfaction and psychological adjustment are interrelated (Gilman and Huebner 2006). Gilman and Huebner (2006) found that adolescents with high life satisfaction had virtually no psychological symptoms, whereas adolescents with low life satisfaction often had mental health problems of a clinical nature. Lewinsohn et al. (1991) found that low life satisfaction predicted subsequent psychological disorders. Suldo and Huebner (2006) found that high life satisfaction was related to better adaptive psychosocial functioning and fewer emotional and behavioral problems. Students with high global life satisfaction tended to experience less intrapersonal distress and a greater sense of hope than students with low life satisfaction (Gilman and Huebner 2006). Thus, high life satisfaction can be regarded as a potential protective factor against hopelessness (Heisel and Flett 2004).

McKnight et al. (2002) showed that adolescents who experienced more stressful events were less satisfied with life and consequently were more likely to have maladaptive internal responses (e.g., anxiety and depression). Sun and Shek (2010, 2012, and 2013) reported that adolescents with high levels of positive youth development were more satisfied with life and had fewer behavioral problems. These findings suggest that life satisfaction functions as a mediator in the relationship between positive youth development and problem behavior. However, there is limited scientific evidence on the mediating role of life satisfaction in the association between perceived school performance and hopelessness. Because school is a means of climbing the social ladder, it influences adolescent life satisfaction and serves as an important determinant of their hope about the future.

### The Present Study

As a transitional stage in life, adolescence is filled with difficulties and challenges (Goldbeck et al. 2007). Adolescents may display different patterns of life satisfaction from adults. However, studies of life satisfaction have mainly focused on adults, even though life satisfaction is regarded as an important construct for people of all age groups (Silva et al. 2014). The same limitation is true for studies on hope, most of which have used adult samples (Gilman and Huebner 2006). To fill this research gap, we recruited a large number of Grade 7 secondary school students (Secondary 1) in 2009 and tracked them until Grade 10 (Secondary 4) to investigate whether adolescent perception of school performance is a determinant of hopelessness, with life satisfaction as a mediating factor. A review of the scientific literature revealed that no studies have yet considered the relationships between perceived school performance, life satisfaction, and hopelessness.

The goal of the study was to explore the developmental pathways of students’ perceptions of school performance, life satisfaction, and hopelessness, and the underlying associations between them. School performance was conceived as perceived academic performance and conduct in school. Several hypotheses were proposed:

- perceived school performance and life satisfaction decrease over time (Hypothesis 1a), whereas hopelessness increases over time (Hypothesis 1b);
- school performance is negatively related to life satisfaction (Hypothesis 2);
- life satisfaction is negatively related to hopelessness (Hypothesis 3);
- school performance is negatively related to hopelessness (Hypothesis 4); and
- life satisfaction mediates the relationship between perceived school performance and hopelessness (Hypothesis 5).

## Methods

### Participants

The data were derived from an on-going 6-year longitudinal study investigating adolescent development in Hong Kong. This study used the data collected at Time 1, Time 2, Time 3, and Time 4. Twenty-eight public secondary schools were randomly selected and invited to participate in this large-scale project. The student participants’ details are reported in Table 1. The data from 2427 students (72.9 % of the 3328 students who completed the first assessment) who completed all four assessments were used in the analyses. Comparison of those participants who only took part in the first assessment and those who completed all assessments revealed that more boys than girls dropped out of this longitudinal study (Pearson *x* ^2^(1) = 41.073, *p* < .001). Regarding the main variables in this study, there was no significant difference between the life satisfaction ratings of drop-out students and retained students (*t* = −1.629, *p* > .05), but drop-out students had significantly lower perceptions of school performance (*t* = −7.104, *p* < .001) and higher levels of hopelessness (*t* = 5.284, *p* < .001).

**Table 1:** Descriptive analyses of the participants across the four time points

Time point	Academic year	Number of participants	Mean age (SD)
Time 1	2009/10	3328	12.59 (.743)
Time 2	2010/11	2905	13.59 (.697)
Time 3	2011/12	2668	14.54 (.680)
Time 4	2012/13	2427	15.49 (.656)

### Procedures

The data collection procedure was the same at each time point. Written informed consent was obtained from the participants’ parents before data collection. Passive informed consent was also obtained from the participants. Trained research assistants were responsible for the administration of the questionnaires. They provided students with standardized instructions (e.g., research objectives, voluntary participation, and data confidentiality), answered their questions about the investigation, and maintained classroom discipline. The same self-report questionnaire (approximately 30 min) was used at each time point.

### Measures

#### Satisfaction with Life Scale (SWLS)

The SWLS (Diener et al. 1985) was used to assess the participants’ global judgment on their quality of life. The SWLS was translated into Chinese and validated by Shek (1992) in Hong Kong. It is a 5-item (e.g., in most ways my life is close to my ideal) scale, measured on a 6-point Likert scale ranging from 1 (*strongly disagree*) to 6 (*strongly agree*). In this study, the score range was between 5 and 30, and Cronbach’s *α* was .849, .873, .872, and .879 at Time 1, Time 2, Time 3, and Time 4, respectively.

#### Chinese Hopelessness Scale (HOPEL)

The original Hopelessness Scale was developed by Beck et al. (1974). It was translated into Chinese with some modifications to measure the sense of hopelessness in Hong Kong (Shek 1997). The 5-item (e.g., the future seems vague and uncertain to me) scale is used to assess individual hopelessness about life, measured on a 6-point Likert scale ranging from 1 (*strongly disagree*) to 6 (*strongly agree*). In this study, total scores ranged from 5 to 30, and Cronbach’s *α* was .851, .862, .875, and .883 at the four assessments, respectively.

#### Academic and School Competence Scale (ASC)

The ASC (Shek and Yu 2012) was used to assess perceived school performance. This construct is operationalized in terms of perceived academic performance and school conduct, assessed by three items (e.g., perceived academic performance compared to schoolmates) using a 5-point Likert scale ranging from 1 (*very poor*) to 5 (*very good*). In this study, the minimum score was 3, the maximum score was 15, and Cronbach’s *α* was .659, .690, .656, and .628 at the four time points, respectively.

### Data Analytic Plan

To avoid the potential problem of collinearity, the tolerance and variance inflation factor (VIF) values were examined. To measure the changes in perceived school performance, life satisfaction, and hopelessness at each time point, one-way repeated measures analyses of variance (ANOVA) were performed in SPSS 21. To test the mediating effect of life satisfaction on the relationship between perceived school performance and hopelessness longitudinally, the two-step modeling approach recommended by Anderson and Gerbing (1988) was adopted. The measurement model and structural model were successively tested using LISREL 8.7 (Jöreskog and Sörbom 2004). Due to the non-normal data distribution, the robust maximum likelihood (RML) estimation method was used. Various indicators were used to assess the goodness of fit of the model, including the comparative fit index (CFI; Bentler 1990), incremental fit index (IFI; Bollen 1989), and non-normed fit index (NNFI; Tucker and Lewis 1973). Goodness-of-fit requires that the values of these indicators are above .95 (Hu and Bentler 1999). Additionally, a value below .80 (Hu and Bentler 1999) for the root mean square error of approximation (RMSEA; Steiger 1990) is considered a good fit. Following the mediation test across the four time points, the mediating effect of life satisfaction was examined at each time point according to Baron and Kenny’s approach (1986). Bootstrapping was performed as this method renders measurement more accurate by replacing the original sample size *n* multiple times (Preacher and Hayes 2008). In this study, the resampling was repeated 10,000 times with the calculation of the bias-corrected (BC) confidence intervals (CIs), suggesting that the lower and upper bounds of the interval would be the 250th and 9751st estimates.

## Results

### Descriptive Statistics and Correlation Analyses

The mean values and standard deviations of perceived school performance, life satisfaction, and sense of hopelessness at the four assessments are reported in Table 2. According to Belsley et al. (1980), a tolerance value below 0.10 or a VIF above 10 implies multi-collinearity. The tolerance values in this study ranged from .854 to .875 and the VIF ranged from 1.171 to 1.142, thus multi-collinearity was not a problem.

**Table 2:** Change trends of perceived school performance, life satisfaction, and hopelessness

	Time 1		Time 2		Time 3		Time 4	
	Mean	SD	Mean	SD	Mean	SD	Mean	SD
ASC	9.51	1.91	9.31	2.00	9.21	1.95	8.85	1.95
LS	19.83	5.37	19.18	5.43	18.90	5.27	18.54	5.27
HL	13.04	5.66	13.40	5.58	13.40	5.51	13.31	5.39

*ASC* academic and school competence, *LS* life satisfaction, *HL* hopelessness

### One-Way Repeated Measures ANOVA

To examine the changes in perceived school performance, life satisfaction, and hopelessness over time, one-way repeated measures ANOVAs were conducted. Perceived school performance (*F* = 220.883, *p* < .001, η^2^ = .083) and life satisfaction (*F* = 119.957, *p* < .001, η^2^ = .047) linearly decreased over time, whereas hopelessness showed a quadratic increasing trend in the same period (*F* = 7.250, *p* < .01, η^2^ = .003). The effect sizes of the differences in the above three variables generally ranged from small to medium. Bonferroni post hoc comparisons indicated that adolescents had better perceptions of their school performance and higher life satisfaction at lower grades than at higher grades. Adolescent hopelessness significantly increased in early adolescence, although this trend remained relatively stable in middle adolescence (see Table 3). The findings provide support for hypotheses 1a and 1b.

**Table 3:** Results of one-way repeated measures ANOVAs on ASC, LS, and HL (Bonferroni post-hoc comparisons)

	F	η^2^	T1–T2	T1–T3	T1–T4	T2–T3	T2–T4	T3–T4
ASC	220.883***	.083	*p* < .001	*p* < .001	*p* < .001	*p* < .05	*p* < .001	*p* < .001
LS	119.957***	.047	*p* < .001	*p* < .001	*p* < .001	*p* < .05	*p* < .001	*p* < .01
HL	7.250**	.003	*p* < .05	*p* < .05	*p* > .05	*p* > .05	*p* > .05	*p* > .05

*ASC* academic and school competence, *LS* life satisfaction, *HL* hopelessness

*T1* Time 1, *T2* Time 2, *T3* Time 3, *T4* Time 4

*** *p* < .001; ** *p* < .01

### Measurement Model

The measurement model included 12 latent variables (perceived school performance, life satisfaction, and hopelessness at the four time points) and 52 observed variables. The measurement model showed a good model fit: *x* ^2^(1208, *n* = 2427) = 11,713, *p* < .001; CFI = .96; IFI = .96; NNFI = .95; and RMSEA = .060 (90 % CI: 0.059–0.061). All of the factor loadings of the observed variables on the latent variables were significant. The factor loadings ranged from .37 to .81 for perceived school performance, .54 to .89 for life satisfaction, and .57 to .91 for hopelessness. Across the four time points, perceived school performance, life satisfaction, and hopelessness were significantly correlated with each other (see Table 4). These findings provide support for hypotheses 2, 3, and 4.

**Table 4:** Correlations between perceived school performance, life satisfaction, and hopelessness

	Time 1			Time 2			Time 3			Time 4		
	1	2	3	4	5	6	7	8	9	10	11	12
*Time 1*												
1. T1 ASC	1											
2. T1 LS	.353	1										
3. T1 HL	−.292	−.301	1									
*Time 2*												
4. T2 ASC	.518	.291	−.234	1								
5. T2 LS	.272	.568	−.292	.377	1							
6. T2 HL	−.227	−.309	.449	−.284	−.378	1						
*Time 3*												
7. T3 ASC	.451	.250	−.202	.573	.290	−.230	1					
8. T3 LS	.229	.483	−.240	.294	.588	−.305	.365	1				
9. T3 HL	−.225	−.256	.405	−.279	−.333	.541	−.321	−.367	1			
*Time 4*												
10. T4 ASC	.389	.226	−.181	.487	.248	−.237	.561	.290	−.302	1		
11. T4 LS	.235	.427	−.200	.267	.495	−.258	.272	.593	−.330	.382	1	
12. T4 HL	−.216	−.205	.359	−.239	−.276	.404	−.279	−.343	.540	−.307	−.378	1

All correlation coefficients are significant at *p* < .001

*ASC* academic and school competence, *LS* life satisfaction, *HL* hopelessness

*T1* Time 1, *T2* Time 2, *T3* Time 3, *T4* Time 4

### Structural Model

**Fig. 1:**
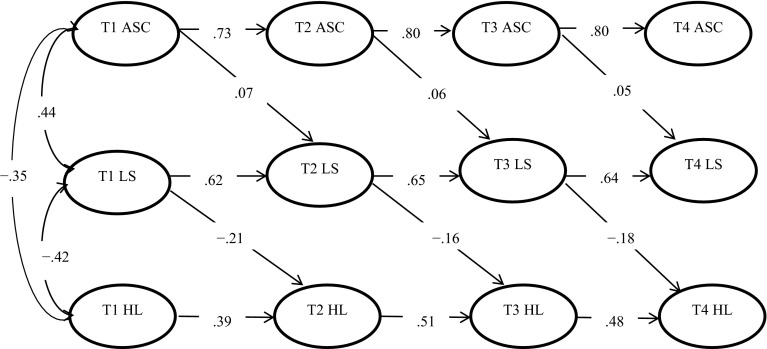
Structural model. All paths are significant (*p* < .05). *ASC* academic and school competence, *LS* life satisfaction, *HL* hopelessness. *T1* Time 1, *T2* Time 2, *T3* Time 3, *T4* Time 4

The structural model (see Fig. 1) fitted the data very well: *x* ^2^(1247, *n* = 2427) = 12,023, *p* < .001; CFI = .96; IFI = .96; NNFI = .95; and RMSEA = .060 (90 % CI: 0.059–0.061). All of the autoregressive paths and hypothesized cross-lag paths were significant, suggesting that life satisfaction mediated the relationship between perceived school performance and hopelessness longitudinally. We also found significant indirect effects of perceived school performance (Time 1) on hopelessness (Time 4) through the mediator of life satisfaction at Time 2 [*β* = −.005, (BC 95 % CI: −.010, −.001)] and Time 3 [*β* = −.007, (BC 95 % CI: −.014, −.002)], after controlling for life satisfaction and hopelessness in the previous wave. Table 5 also shows that the mediating role of life satisfaction was established in all four assessments. “Zero” was not included in the bias-corrected confidence intervals, indicating that the indirect effects were significant. After controlling for life satisfaction, the direct effects of perceived school performance on hopelessness were still significant at each time point (*p*s < .001). This result implies that life satisfaction functioned as a partial mediator of the relationship between perceived school performance and hopelessness. Overall, adolescent life satisfaction mediated the relationship between perceived school performance and hopelessness in both a longitudinal and a cross-sectional manner. The findings provide support for Hypothesis 5.

**Table 5:** Mediating effect of life satisfaction in the relationship between perceived school performance and hopelessness across the four time points

Time	*a* path	*b* path	*c* path	*c′* path	*ab* path	BC 95 % CI	
						Lower	Upper
Time 1	.353	−.226	−.292	−.213	−.080	−.101	−.062
Time 2	.377	−.316	−.284	−.165	−.119	−.142	−.098
Time 3	.365	−.288	−.321	−.216	−.105	−.128	−.085
Time 4	.382	−.305	−.307	−.191	−.117	−.138	−.096

All coefficients are standardized and significant at *p* < .001

*a* path, IV to mediator; *b* path, direct effects of mediator on DV; *c* path, total effect of IV on DV; *c′* path, direct effect of IV on DV; *ab* path, indirect effects of IV on DV through mediator; BC CI, bias corrected confidence intervals

## Discussion

In this study, we investigated the developmental trends of perceived school performance, life satisfaction, and hopelessness in adolescents using 4-year longitudinal data. We also explored the mediating effect of life satisfaction on the relationship between perceived school performance and hopelessness. As expected, we found that adolescents’ perceptions of their school performance and life satisfaction dropped significantly from Secondary 1 to Secondary 4, whereas hopelessness increased over the same period (Hypotheses 1a and 1b). School performance was significantly related to life satisfaction (Hypothesis 2), life satisfaction was significantly related to hopelessness (Hypothesis 3), and school performance was significantly related to hopelessness (Hypothesis 4). Life satisfaction functioned as a cross-sectional and longitudinal mediator in the association between perceived school performance and hopelessness (Hypothesis 5). These findings are pioneering in both the Western and Chinese scientific literature.

As the depth and breadth of knowledge advances with each school grade, achieving good school performance becomes more difficult for adolescent students. As a result, students’ perceptions of their school performance decline as the demands on them at school increase. This supports the finding of Leung et al. (2004) that adolescents’ perceptions of school performance and academic self-image declined after they entered junior secondary school. It also provides further evidence that adolescents are more likely to be involved in school misconduct (e.g., school truancy) as they get older (Roeser and Eccles 1998; Shek and Lin 2014).

In addition to the decline in perceived school performance, adolescent life satisfaction also showed a decreasing trend and their sense of hopelessness increased accordingly. The decrease in life satisfaction and increase in hopelessness experienced during adolescence lend credence to the findings of a European study, in which both male and female adolescents showed a significant declining trend in personal well-being (Michel et al. 2009). There are two possible explanations for this trend. One possibility is that adolescence, as an important developmental stage between childhood and adulthood, is filled with many physical and psychosocial changes that generate emotional upheaval and increase emotional distress (Yeo et al. 2007). As competition is fierce in the contemporary world and life is more complicated (e.g., parental marital disruption and economic instability), adolescents have to struggle with different kinds of challenges from those they face in childhood. Another possibility is that older adolescents with better cognitive abilities tend to have more realistic perceptions of the world (Shek and Liu 2014). This implies that adolescents become more mature and look at things from different angles, which gives them a clearer understanding of their current situation and future development.

The significant association between perceived school performance and life satisfaction in this study supports Hypothesis 2 and is congruent with the previous finding that good school experiences lead to high life satisfaction (Gilman and Huebner 2006). If people appraise their life events as undesirable, they will become dissatisfied with life (Myers and Diener 1995). In this case, compared with students with more favorable perceptions of their school performance, students with less favorable perceptions were more likely to be dissatisfied with life. The finding that life satisfaction contributed to a sense of hopelessness in this study provides support for Hypothesis 3. It is also in line with the finding that life satisfaction and negative affect are negatively correlated (Garcia and Moradi 2013; Huebner and Dew 1996; Orkibi et al. 2014) and that a low level of life satisfaction significantly predicts mental disorders (Gilman and Huebner 2003).

The mediating role of life satisfaction is another important finding of this study as it provides insight into the associations between perceived school performance, life satisfaction, and hopelessness (Hypothesis 5). The result implies that perceived school performance influences hopelessness through the mediating mechanism of life satisfaction. In other words, if adolescents have poorer perceptions of their school performance, they are more likely to have low life satisfaction, which further leads to a greater sense of hopelessness. The total effect of perceived school performance was significant in predicting adolescents’ sense of hopelessness. This supports previous studies that found self-perceptions of school performance to influence student well-being (Van Petegem et al. 2007), and that students’ hopeful thinking originated from their perceived capabilities (Snyder et al. 1997). Because the importance of school success in Chinese society is undisputed (Li et al. 2010), students’ sense of hope is influenced to a great extent by their perceived school performance (Chang 1998). Due to the dominant status of study in adolescent life, if students have poorer perceptions of their school performance, they are more likely to feel hopeless (Schutz and Pekrun 2007) because negative emotions increase in response to stressful life events (Suh et al. 1998).

We found that the direct effect of perceived school performance on hopelessness was significant even after controlling for life satisfaction. This result suggests that the mediating effect of life satisfaction is only partial in nature. Perceived school performance has a direct effect on hopelessness besides the indirect effect, possibly because school performance is one of the biggest stressors for adolescents as it can determine their future (Bray and Kwok 2003). Therefore, perceived school performance influences not only life satisfaction, but also self-expectations and hope for the future.

The developmental trends of perceived school performance, life satisfaction, and hopelessness, together with the mediating role of life satisfaction discovered in this study, provide insight for prevention and intervention. From early to middle adolescence, adolescents face many new difficulties that may lead to a gradual decrease in perceived school performance, life satisfaction, and sense of hope. Thus, it would be helpful to initiate a target program to facilitate adolescent students in developing positive and reasonable perceptions of their school performance and appropriate estimations of the role of school performance in their lives. In particular, looking at students’ strengths in different domains of their school life would help them to develop more balanced evaluations of themselves. Sun and Shek (2010, 2012) showed that high life satisfaction could prevent adolescents from developing behavioral problems.

This study represents an initial attempt to explore the development of adolescents’ perceptions of school performance, life satisfaction, and hopelessness via a 4-year longitudinal study in China. It is a pioneering effort in the Chinese and international literature, but several limitations should be noted. First, although 72.9 % of the students (*n* = 2427) completed the four assessments, 901 students (i.e., 27.1 % of the participants who completed the first assessment) dropped out for various reasons (e.g., leaving their current schools). Second, we measured perceived rather than actual school performance because students’ academic and conduct grades are confidential. This precluded us from understanding whether actual school performance plays the same role as perceived school performance in contributing to hopelessness. Third, we measured global life satisfaction rather than life satisfaction in specific domains. Satisfaction with specific areas of life (e.g., school life) could be measured to explore whether perceived school performance has a greater influence on school satisfaction than global life satisfaction. Fourth, as self-report measures were used, an alternative explanation based on shared-method variance cannot be totally dismissed. There is no doubt that information from parents, teachers, and peers should be taken into account to reduce the single-reporter bias and increase the credibility of the findings. Last but not least, although this study used a large sample, researchers should remain cautious in generalizing the findings to other cultures and contexts.

Despite these limitations, this pioneering study enriches the literature on the developmental trajectory of adolescents’ perceived school performance, life satisfaction, and hopelessness in the high school period and clarifies their inter-relationships, particularly within Chinese culture. The accumulation of knowledge about adolescents’ perceptions of school performance, life satisfaction, and hopelessness can help youth workers and allied professionals to design better intervention and evaluation programs to promote adolescents’ well-being in school practice. The findings of the present study suggest that changing students’ perceptions of their school performance and promoting their satisfaction with life is helpful in reducing their sense of hopelessness. These suggestions are consistent with the intervention foci of cognitive and cognitive-behavioral approaches in youth counseling.
